# Seroepidemiology of enterovirus D68 infection in China

**DOI:** 10.1038/emi.2017.14

**Published:** 2017-05-10

**Authors:** Zichun Xiang, Linlin Li, Lili Ren, Li Guo, Zhengde Xie, Chunyan Liu, Taisheng Li, Ming Luo, Gláucia Paranhos-Baccalà, Wenbo Xu, Jianwei Wang

**Affiliations:** 1 MOH Key Laboratory of Systems Biology of Pathogens and Christophe Mérieux Laboratory, IPB, CAMS-Fondation Mérieux, Institute of Pathogen Biology (IPB), Chinese Academy of Medical Sciences (CAMS) & Peking Union Medical College, Beijing 100730, China; 2 Collaborative Innovation Center for Diagnosis and Treatment of Infectious Diseases, Hangzhou 310003, China; 3 Beijing Children’s Hospital Affiliated to Capital University of Medical Sciences, Beijing 100045, China; 4 Peking Union Medical College Hospital, Beijing 100005, China; 5 Beijing Center for Diseases Control and Prevention, Beijing 100013, China; 6 Fondation Mérieux, Lyon 69365, France; 7 WHO WPRO Regional Polio Reference Laboratory and Ministry of Health Key Laboratory for Medical Virology, National Institute for Viral Disease Control and Prevention, Chinese Center for Disease Control and Prevention, Beijing 102206, China

**Keywords:** enterovirus D68, epidemic, neutralizing antibody, seroprevalence

## Abstract

Human enterovirus 68 (EV-D68) is a rarely reported virus that has been linked to respiratory disease. In recent years, reports about EV-D68 infection have markedly increased worldwide. However, the epidemiological features of this emerging infection are not well understood. To evaluate the emerging EV-D68 epidemic, we isolated the circulating viral strain and investigated the seroprevalence of neutralizing antibodies (NAbs) in Beijing between 2004 and 2011. We found that the titers of EV-D68 NAbs were generally low in all age groups in sampled populations in 2004 but significantly higher in 2009. From 2007 to 2011, the NAbs against EV-D68 significantly increased over time. These findings indicate that EV-D68 has spread widely in the Chinese population in recent years, although only a limited number of cases were reported.

## INTRODUCTION

Enterovirus D68 (EV-D68) belongs to the species Enterovirus D within the *Enterovirus* genus. The biological properties of acid lability and a lower optimum growth temperature suggest that EV-D68 is similar to human rhinoviruses (HRVs) and is a respiratory tract pathogen.^1, 2^ As a non-enveloped, positive-sense, single-stranded RNA virus, EV-D68 genome contains a single open reading frame coding for a polyprotein, which is ultimately cleaved into four viral capsid proteins VP1–VP4 and seven non-structural proteins 2A–2C, 3A–3D by its proteases 2A and 3C.^3^ VP1, VP2 and VP3 harbor the epitopes for neutralizing antibodies (NAbs).^4^

EV-D68 has been rarely detected since its first identification in 1962.^2^ Based on the enterovirus surveillance data, only 36 cases were identified from 1970 to 2005 in the USA.^5^ However, EV-D68 infection in patients with respiratory tract infections (RTIs) increased markedly in recent years worldwide possibly due to the viral genome variation.^6, 7, 8, 9, 10, 11, 12^ Particularly, EV-D68 infections spread widely in 2014 in the United States causing outbreaks in most states. More than 1000 cases were reported during the epidemic.^13^ In addition to respiratory illness, EV-D68 infection is also associated with neurological complications in the USA.^14^ This has raised a public health concern. To date, no specific drugs and vaccines specifically targeting EV-D68 are available.

The earliest EV-D68 infection in China was identified in 2006.^9^ More cases were detected in different geographical locations in subsequent years by different groups.^15, 16, 17, 18, 19^ In contrast to the prevalence observed in the United States, there was no outbreak noted in China. The reason for this observation is unclear. Besides viral factors, herd immunity, particularly the pre-existing NAbs in a population, may influence the spread of a virus.^20^ As an imprint of the immunoresponse, NAbs in specific populations can not only trace the history of infection but also predict the susceptibility to a certain pathogen.^21^ Because EV infections are usually asymptomatic or mild,^22^ the data obtained from RTI patients seeking medical services could grossly underestimate the actual incidence and prevalence. To reveal an accurate epidemiological picture of an emerging virus infection, serological investigations based on NAb detection in the general population are therefore of particular importance to assess the prevalence and the transmission potential of EV-D68 for taking public measures to prevent this emerging epidemic.^22^

Circulating EV-D68 strains can be divided into three clades: clades A–C.^8^ Based on phylogenetic analysis, from August 2006 to August 2011, strains of clade A were predominant (90.9%) and clade B strains emerged in October 2011 in China.^19^ In this study, we isolated an EV-D68 strain in China of clade A and set up microneutralization assays (MNAs) using sera collected before 2011 to reveal an accurate epidemiological picture of this emerging infection in Beijing, China.

## MATERIALS AND METHODS

### Sera

Horse EV-D68 (Fermon, Manassas, VA, USA) antiserum was purchased from American Type Culture Collection (ATCC, Manassas, VA, USA). Pooled horse antisera against the most frequently isolated echoviruses and coxsackieviruses (National Institute of Public Health and the Environment (RIVM), The Netherlands) were provided by the World Health Organization (WHO).^23^

Serum specimens were collected from 393 healthy individuals aged 0–93 years seen for routine health check-ups in 2004 and 2009 (Table 1). In addition, serum samples were collected from 169 children (0.6–177 months) with a primary diagnosis of lower RTIs (LRTIs) including bronchitis, bronchiolitis and pneumonia upon admission to the Beijing Children’s Hospital in 2007, 2009 and 2011. Serum samples also were collected from 374 adults (16–59 years) with acute RTIs (369 (98.7%) patients with upper respiratory tract infections (URTIs) and 5 (1.3%) patients with LRTIs) at the time of their admission to the Fever Clinic Department of the Peking Union Medical College Hospital (PUMCH) in Beijing, China (Table 1). As EV-D68 circulates in summer and autumn,^19, 24^ adult’s sera were all collected from August to October of the selected years.

Common respiratory viruses (RVs) including influenza viruses (A, B and C), human parainfluenza viruses (1–4), respiratory syncytial virus, human coronaviruses (229E, NL63, HKU1 and OC43), metapneumovirus, adenovirus and HRVs were screened in the respiratory specimens of these patients by multiplex RT-PCR, single RT-PCR or PCR assays as described elsewhere.^25^ EVs were amplified 350–400 nt of the viral protein 1 gene by RT-PCR^26^ and verified the findings by sequence analysis.^9, 19^

All samples were collected after obtaining informed consent either from the individuals or from the individual’s guardians. This study was approved by the ethical review committee of the Institute of Pathogen Biology, Chinese Academy of Medical Sciences. The sera were separated immediately after collection, stored at −80 °C and inactivated at 56 °C for 30 min before use.

### Viruses

The EV-D68 prototype strain, Fermon (GenBank accession no. AY426531) was purchased from ATCC. We isolated an EV-D68 strain from the nasopharyngeal aspirates (NPAs) of an EV-D68-positive, 32-month-old male patient with pneumonia. This patient displayed a 2-day long cough, fever (the highest temperature: 38.2 °C), sneezing and runny nose. The NPAs were first inoculated into a monolayer of H1-HeLa cells (ATCC CRL-1958). After 1 h adsorption at 33 °C in a humidified 5% CO_2_ atmosphere, the NPAs were removed and the treated H1-HeLa cells were further incubated at 33 °C in DMEM medium supplemented with 2% bovine fetal serum. The cultures were held for five to seven days and examined periodically for viral cytopathic effects (CPEs).^27^ The negative cultures in the first passage were passed blindly to new cell cultures and examined for CPE. When CPE appeared, the isolate was first identified by negative-staining electron microscopy and characterized by MNAs using horse EV-D68 (Fermon) antiserum and a standard pool of EV typing antisera (RIVM, The Netherlands), then by western blot analysis using a specific antibody of VP1^28^ and complete genome sequencing. The isolated virus was designated as EV-D68/Beijing/2008/01. Phylogenetic analysis indicates that EV-D68/Beijing/2008/01 belong to clade A.

### Neutralization test

MNAs were performed in accordance with the WHO standard procedure for poliovirus.^29^ Serum sample dilutions of 1:8 to 1:2048 were assayed, and each dilution was tested in quadruplicate. Twenty-five microliters of 100 tissue culture infective dose (TCID_50_) of virus was mixed with 25 μL of the appropriate serum dilution, and then incubated for 1 h at 33 °C in a CO_2_ incubator to allow the antibodies bind to the virus. After the incubation period, 50 μL of the serum–virus suspension was added to monolayers of H1-HeLa cells and incubated for 1 h at 33 °C in a 5% CO_2_ incubator before washing and reincubating with minimal essential media with 2% FBS. Cell controls (without virus), virus controls (without serum) and virus back titration were included in each batch. The horse EV-D68 (Fermon) antiserum was selected as positive control in the MNAs. The NAb titers were determined at the time when CPE was observed in virus controls under an inverted microscope and calculated by the Reed–Muench method.^30^

### Statistical analysis

The antibody titers for EV-D68 and other EVs are presented as geometric mean titer (GMT) and the 95% confidence interval (95% CI), calculated from log-transformed titers using *t* distributions. For comparisons of GMTs between different viruses, age groups and study years, one-way analysis of variance on log-transformed titers were used if normality and homogeneity of variance were assumed for log-transformed data; otherwise a nonparametric Kruskal–Wallis test would be used. *P*-values <0.05 were considered statistically significant. All statistical analyses were conducted using R version 2.15.3

## RESULTS

### EV-D68 isolation

**Figure 1:**
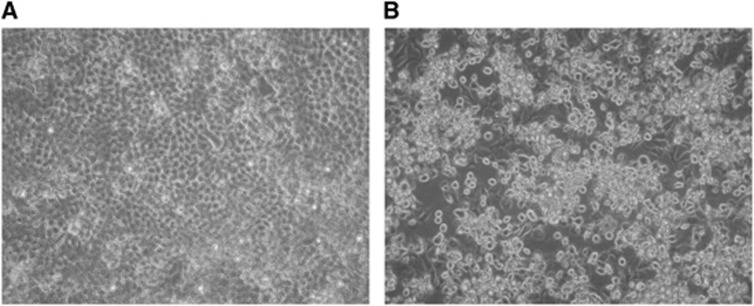
Cytopathic effects observed in H1-HeLa cells ( × 20). (**A**) Untreated control. (**B**) Cells infected with the isolate.

We visualized typical picornavirus-like CPE after 3 days of the second passage of the inoculation on H1-HeLa cells. The cells appeared rounding and sloughing under a light microscope (Figure 1).^27^ The cell culture supernatants contained spherical particles with a diameter of about 30 nm under negative-staining electron microscopy.

We then examined the cross-reaction between the isolate and the reference EV antisera. The MNAs showed that the isolate could not be neutralized by any of the pooled EV typing antisera (RVIM, The Netherlands) but could only be neutralized by EV-D68 (Fermon) antiserum (neutralizing titer: 64). Western blot analysis of the pellets using antibodies against VP1 of EV-D68 and EV-A71 showed that only the anti-EV-D68 antibody hybridized with inoculated cells. These findings indicate that clinical strain of EV-D68 was isolated successfully. We designated the strain as EV-D68/Beijing/2008/01.

Based on the complete genome sequence, the viral genome is 7348 nt in length (GenBank accession no. KF726085) with 88.2% identity to that of the Fermon strain (7367 nt; GenBank accession no. AY426531), with a 24 nt deletion at nt positions 681–704 and an additional 3 nt deletion at positions 2806–2808. The sequence had a 94.5% identity with that of NYC403 (7341 nt; GenBank accession no. JX101846), the representative strain of clade A.^8^ This result suggests that EV-D68/Beijing/2008/01 is a strain of clade A.

### NAbs against EV-D68/Beijing/2008/01

**Figure 2:**
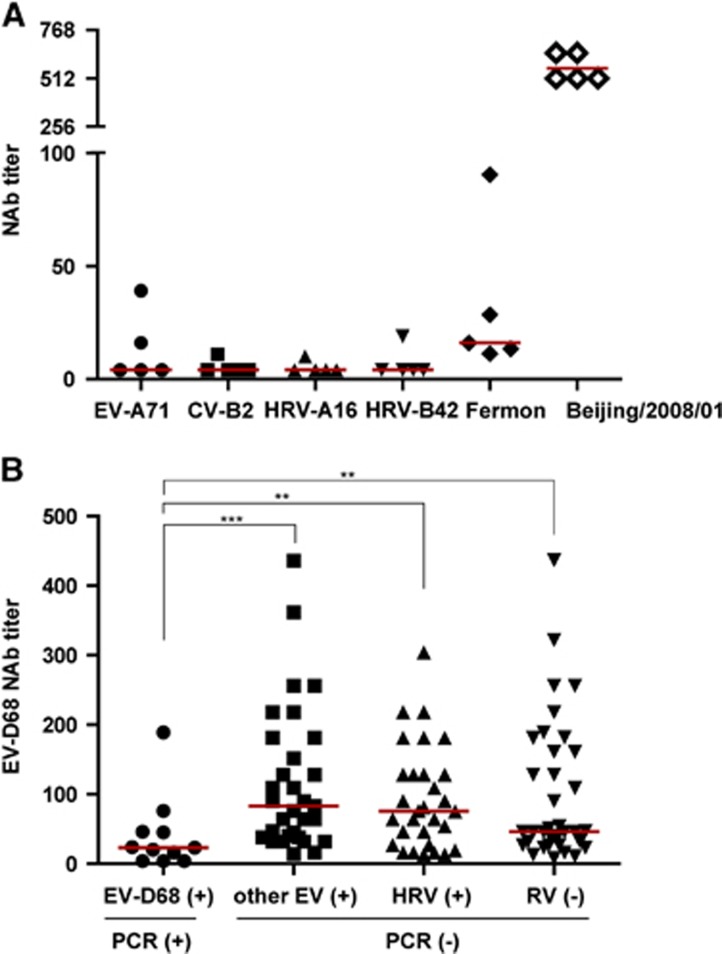
Specificity of NAbs against EV-D68. (**A**) Different titers of NAbs against EV-A71, CV-B2, rhinovirus A16 (HRV-A16), HRV-B42, Fermon or EV-D68/Beijing/2008/01 in the same sera. Microneutralization assays were performed for five sera using EV-A71, CV-B2, HRV-A16, HRV-B42, Fermon and EV-D68/Beijing/2008/01, respectively. (**B**) Microneutralization assays were performed using EV-D68/Beijing/2008/01, and sera of adults whose nasal and throat swabs were EV-D68 positive and negative were analyzed by RT-PCR. Compared with EV-D68-positive group, ***P<0.001; **P<0.01. Human rhinovirus, HRV; neutralizing antibodies, NAbs; respiratory virus, RV. Red lines indicate median. The NAb titers are graphed as a scatter plot to show the NAb titer of each serum.

As EV-D68 is an emerging pathogen, there are no commercial antibodies available against currently circulating strains. So we used antiserum against Fermon strain as positive control. We also performed MNAs using sera which had high neutralizing titer of NAbs against EV-D68/Beijing/2008/01. These sera also could neutralize Fermon strain but had low titers (Figure 2A).

To further demonstrate whether NAbs could protect people from EV-D68 infection, we compared the NAb levels between the acute phase sera from adults who were EV-D68 positive, other EV-positive but EV-D68-negative, HRV-positive or respiratory viruses-negative as detected by RT-PCR. We found that the GMT of EV-D68-positive adults was 21 (95% CI, 9–49), which was significantly lower than that of the group of other EV-positives (82) (95% CI, 59–113), HRV-positives (65) (95% CI, 46–92), and respiratory virus-negatives (60) (95% CI, 43–84; Figure 2B). This result indicates that NAbs against EV-D68 could protect people from EV-D68 infection. Although other EVs and HRVs belong to the same genus as EV-D68, these results suggest that high titer anti-EV-D68 NAbs do not protect people from other EVs or rhinovirus infections.

### NAbs against EV-D68 in 2004 and 2009

**Figure 3:**
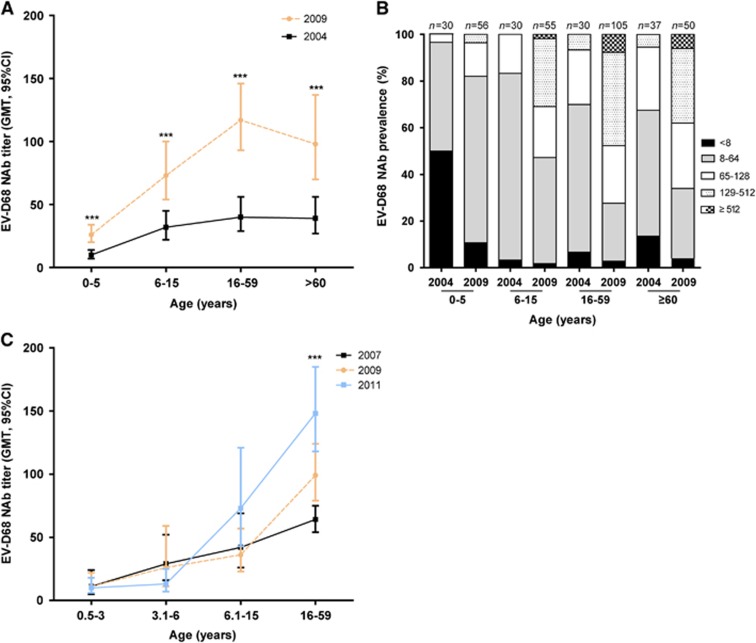
Increase in the titers of neutralizing antibodies against EV-D68 after 2006. (**A**) Anti-EV-D68 neutralizing antibodies (NAbs) in sera collected from healthy individuals in 2004 and 2009. NAb titers are graphed as the geometric mean titer (GMT) with a 95% confidence interval (95% CI). ***Compared with the NAbs in 2004, *P*<0.001. (**B**) Prevalence of EV-D68 NAbs in different age groups of healthy individuals. (**C**) NAbs against EV-D68 in patients with respiratory tract infections (RTIs) from 2007 to 2011. NAb titers were graphed as the GMT with a 95% CI. ***Compared with the NAbs in 2007 and 2009, *P*<0.001.

Because most EV-D68 infections in China were detected in our previous study in 2006,^9^ we tested sera collected from healthy individuals prior to and after 2006 (2004 and 2009, respectively) for NAb detection. We divided the samples into four age groups: preschoolers (≤5 years); school-aged children (6–15 years); adults (16–59 years); elderly (≥60 years). Our data indicate that the GMTs of EV-D68 NAbs increased with age, peaking at adults, but declined in the elderly group (Figure 3A). The GMTs of NAb were generally low in 2004, but were significantly higher in 2009 in each age group.

To characterize the immune level precisely, we defined five NAbs titer ranges: no NAbs (<8), low level (8–64), moderate level (65–128), high level (129–512) and very high level (>512). We found that while low-level NAbs dominated in all age groups in 2004 (Figure 3B), moderate- and high-level NAbs dominated in all age groups in 2009 except in preschoolers. These results suggest an increasing spread of EV-D68 after 2004, which led to the boost of the EV-D68 NAb titer.

### Trend of EV-D68 NAbs from 2007 to 2011

As anti-EV-D68 NAbs in 2009 were significantly higher than those in 2004, we then traced the temporal dynamics of NAbs in the Chinese population in recent years. As we could only get samples from healthy individuals in 2004 and 2009 and the NAbs in RTI patients could block EV-D68 replication, we used sera collected from RV-negative RTI patients as surrogates to perform MNAs. Children were divided into three age groups: 0.5–3 years, 3.1–6 years and 6.1–15 years. Our results showed that in each year, just like in healthy individuals, the GMTs of EV-D68 NAbs in RTI patients also increased with age. From 2007 to 2011, the NAbs increased over time in adults (Figure 3C), suggesting that EV-D68 circulated widely in China after 2006 even though only a limited number of EV-D68-positive patients were detected. From 2007 to 2011, we detected respiratory specimens from 3030 children with a primary diagnosis of LRTIs and 7697 adults with acute RTIs, only 4 children (1–32 months) and five adults (29–34 years) were EV-D68-positive.

## DISCUSSION

Compared with the prototype Fermon strain, the clinical isolates had sequence diversities in the residues flanking the putative antigenic sites, which resulted in differences in neutralization titers for the same antiserum.^31^ Therefore, clinical isolates other than Fermon should be used to evaluate the seroprevalence to EV-D68 in the general population. In this study, we isolated virus from clinical samples and identified it using EV-D68 (Fermon) antiserum and pooled EV typing antisera (RVIM, The Netherlands). Phylogenetic and complete genome analysis demonstrated that this isolate belonged to clade A, which was the main clade circulating in China before 2011. We hence used this isolate to test seroprevalence to EV-D68 in China before 2011.

Our seroprevalence data show that the anti-EV-D68 NAb level was generally low in the Chinese population in 2004. Indeed, the GMT in adults (40) (95% CI, 29–56) was similar to that in the Finnish population in 2002 (Fermon strain, 44.5).^32^ Although a different strain of EV-D68 was used in the Finnish study compared to our study, the low levels of EV-D68 NAbs in the general population in 2004 and the emerged multiple clades of the virus could explain why EV-D68 spread worldwide rapidly in recent years.

The apparent rise in the titer of NAbs against EV-D68 from 2007 to 2011 in adults indicates that NAbs were boosted by infections of EV-D68. Yet limited EV-D68-positive cases were detected in RTI adults in China during this period.^9, 15^ As these specimens were all collected from patients who were seeking medical service at hospitals, the low detection rate of EV-D68 in the general population suggests that EV-D68 mainly causes mild or asymptomatic infections in adults, which do not necessitate a visit to a doctor.

The titer of NAbs in children increased with age. The GMTs were higher in the group of 6.1–15 years than in the group of 0.5–6 years. But even in the group of 6.1–15 years, the GMT (45; 95% CI, 34–59) still had no statistic difference compared to the group of EV-D68-positive adults (21; 95% CI, 9–49). In contrast to the increase year by year in adults, we did not find a significant increase year by year in children. Even in the group of 6–15 years, the GMT in 2011 had no statistic difference compared to that in 2007 or 2009. The reason for the low titer of NAbs in children is not clear. This phenomenon is noteworthy for the designer of vaccine.

In contrast to the large-scale outbreak of EV-D68 that occurred in 2014 in the USA, only very few EV-D68 cases were reported in China in that year.^19^ The high titers of NAbs existing in the Chinese population might contribute to the low prevalence in China. According to our data, the NAbs indeed could block the EV-D68 replication. Seroprevalence of NAbs against EV-D68 increased rapidly to very high level in Chinese population. However, this hypothesis warrants further investigations to compare the seroprevalence in different countries at the same time points.

In our study, only acute phase sera were collected from EV-D68-positive patients and no paired convalescent sera were available to demonstrate seroconversion after infection. We thus compared only the NAbs in acute phase patients who were positive or negative for EV-D68. Nevertheless, the significant difference between these two groups suggests that high titers of NAbs may protect people from EV-D68 infection.

In conclusion, our data indicate that this emerging virus has spread rapidly in China in recent years, resulting in increased levels of NAbs in the general population, although only limited cases were reported.

## Figures and Tables

**Table 1:** Characteristics of sera used in this study (*n*=936)

**Population**	**Collection year**	**Age (years)**	**GMT (95% CI)**	**Number**
*Children with acute lower respiratory infections* a, b				
RVs (−)	2007	0.5–3	11 (5–24)	14
		3.1–6	29 (16–52)	9
		6.1–15	42 (26–69)	18
	2009	0.5–3	11 (6–22)	20
		3.1–6	26 (11–59)	17
		6.1–15	36 (23–57)	37
	2011	0.5–3	10 (6–18)	18
		3.1–6	13 (7–25)	18
		6.1–15	73 (43–121)	18

*Adults with acute respiratory infections* a, c				
EV-D68 (+)			21 (9–49)	11
Other EVs (+)^d^			82 (59–113)	31
Rhinovirus (+)	2006	16–59	65 (46–92)	30
RVs (−)			60 (43–84)	37
RVs (−)	2007		64 (54–75)	96
	2009		99 (79–124)	87
	2011		148 (118–185)	82

*Healthy individuals*				
	2004	0–5	10 (7–14)	30
		6–15	32 (22–45)	30
		16–59	40 (29–56)	30
		≥60	39 (27–56)	37
	2009	0–5	26 (20–34)	56
		6–15	73 (54–100)	55
		16–59	117 (93–146)	105
		≥60	98 (70–137)	50

Abbreviations: enterovirus, EV; geometric mean titer, GMT; respiratory virus, RV.

a RVs, including respiratory syncytial virus, influenza viruses, parainfluenza viruses 1–4, human metapneumovirus, rhinoviruses, EVs, adenoviruses and coronaviruses (229E, OC43, NL63 and HKU1), were screened in the corresponding respiratory specimens of these patients.

b Samples were taken from Beijing Children’s Hospital.

c Samples were taken from Peking Union Medical College Hospital.

d Including 2 coxsackievirus (CV) A10, 1 CVA12, 15 CVA21, 4 CVA9, 4 CVB1, 1 CVB3, 2 CVB5, 1 echovirus (E) 25 and 1 E3 positive.

## References

[bib1] Oberste MS, Maher K, Schnurr D et al. Entervirus 68 is associated with respiratory illness and shares biological features with both the enteroviruses and rhinoviruses. J Gen Virol 2004; 85: 2577–2584. 15302951 10.1099/vir.0.79925-0

[bib2] Schieble JH, Fox VL, Lennette EH. A probable new human picornavirus associated with respiratory diseases. Am J Epidemiol 1967; 85: 297–310. 4960233 10.1093/oxfordjournals.aje.a120693

[bib3] Xiang Z, Wang J. Enterovirus D68 and human respiratory infections. Semin Respir Crit Care Med 2016; 37: 578–585. 27486738 10.1055/s-0036-1584795 PMC7171721

[bib4] Liu Y, Sheng J, Fokine A et al. Structure and inhibition of EV-D68, a virus that causes respiratory illness in children. Science 2015; 347: 71–74. 25554786 10.1126/science.1261962 PMC4307789

[bib5] Khetsuriani N, Lamonte–Fowlkes A, Oberst S et al. Enterovirus surveillance—United States, 1970–2005. MMWR Surveill Summ 2006; 55: 1–20. 16971890

[bib6] Imamura T, Fuji N, Suzuki A et al. Enterovirus 68 among children with severe acute respiratory infection, the Philippines. Emerg Infect Dis 2011; 17: 1430–1435. 21801620 10.3201/eid1708.101328 PMC3381551

[bib7] Centers for Disease Control and Prevention (CDC). Clusters of acute respiratory illness associated with human enterovirus 68–Asia, Europe, and United States, 2008–2010. MMWR 2011; 60: 1301–1304. 21956405

[bib8] Tokarz R, Firth C, Madhi SA et al. Worldwide emergence of multiple clades of enterovirus 68. J Gen Virol 2012; 93: 1952–1958. 22694903 10.1099/vir.0.043935-0 PMC3542132

[bib9] Xiang Z, Gonzalez R, Wang Z et al. Coxsackievirus A21, enterovirus 68, and acute respiratory tract infection, China. Emerg Infect Dis 2012; 18: 821–824. 22516379 10.3201/eid1805.111376 PMC3358056

[bib10] Meijer A, Benschop KS, Donker GA et al. Continued seasonal circulation of enterovirus D68 in The Netherlands, 2011-2014. Euro Surveill 2014; 19: 20935. 25358039 10.2807/1560-7917.es2014.19.42.20935

[bib11] Imamura T, Oshitani H. Global reemergence of enterovirus D68 as an important pathogen for acute respiratory infections. Rev Med Virol 2015; 25: 102–114. 25471236 10.1002/rmv.1820 PMC4407910

[bib12] Bragstad K, Jakobsen K, Rojahn AE et al. High frequency of enterovirus D68 in children hospitalised with respiratory illness in Norway, autumn 2014. Influenza Other Respir Viruses 2015; 9: 59–63. 25534826 10.1111/irv.12300 PMC4353317

[bib13] Centers for Disease Control and PreventionEnterovirus D68 in the United States, 2014. CDC: Atlanta. 2014. Available at http://www.cdc.gov/non-polio-enterovirus/outbreaks/EV-D68-outbreaks.html (accessed 22 December 2014).

[bib14] Holm-Hansen CC, Midgley SE, Fischer TK. Global emergence of enterovirus D68: a systematic review. Lancet Infect Dis 2016; 16: e64–e75. 26929196 10.1016/S1473-3099(15)00543-5

[bib15] Lu QB, Wo Y, Wang HY et al. Detection of enterovirus 68 as one of the commonest types of enterovirus found in patients with acute respiratory tract infection in China. J Med Microbiol 2014; 63: 408–414. 24324030 10.1099/jmm.0.068247-0

[bib16] Zhang T, Ren L, Luo M et al. Enterovirus D68-associated severe pneumonia, China, 2014. Emerg Infect Dis 2015; 21: 916–918. 25897574 10.3201/eid2105.150036 PMC4412250

[bib17] Xiao Q, Ren L, Zheng S et al. Prevalence and molecular characterizations of enterovirus D68 among children with acute respiratory infection in China between 2012 and 2014. Sci Rep 2015; 5: 16639. 26568267 10.1038/srep16639 PMC4644992

[bib18] Zhang T, Li A, Chen M et al. The respiratory infections associated with enterovirus D68 from 2011 to 2015 in Beijing, China. J Med Virol 2016; 88: 1529–1534. 26896830 10.1002/jmv.24505 PMC7166988

[bib19] Xiang Z, Xie Z, Liu L et al. Genetic divergence of enterovirus D68 in China and the United States. Sci Rep 2016; 6: 27800. 27278628 10.1038/srep27800 PMC4899779

[bib20] Zinkernagel RM, LaMarre A, Ciurea A et al. Neutralizing antiviral antibody responses. Adv Immunol 2001; 79: 1–53. 11680006 10.1016/S0065-2776(01)79001-3 PMC7130890

[bib21] Wang X, Xing M, Zhang C et al. Neutralizing antibody responses to enterovirus and adenovirus in healthy adults in China. Emerg Microbes Infect 2014; 3: e30. 26038738 10.1038/emi.2014.30 PMC4051363

[bib22] Weber B, Rabenau H, Cinatl J et al. Quantitative detection of neutralizing antibodies against polioviruses and non-polio enteroviruses (NPEV) using an automated microneutralization assay: a seroepidemiologic survey. Zentralbl Bakteriol 1994; 280: 540–549. 8061416 10.1016/s0934-8840(11)80515-3

[bib23] Hu L, Zhang Y, Hong M et al. Phylogenetic evidence for multiple intertypic recombinations in enterovirus B81 strains isolated in Tibet, China. Sci Rep 2014; 4: 6035. 25112835 10.1038/srep06035 PMC4129410

[bib24] Shaw J, Welch TR, Milstone AM. The role of syndromic surveillance in directing the public health response to the enterovirus D68 epidemic. JAMA Pediatr 2014; 168: 981–982. 25259931 10.1001/jamapediatrics.2014.2628

[bib25] Ren L, Gonzalez R, Wang Z et al. Prevalence of human respiratory viruses in adults with acute respiratory tract infections in Beijing, 2005-2007. Clin Microbiol Infect 2009; 15: 1146–1153. 19456830 10.1111/j.1469-0691.2009.02746.x PMC7129754

[bib26] Nix WA, Oberste MS, Pallansch MA. Sensitive, seminested PCR amplification of VP1 sequences for direct identification of all enterovirus serotypes from original clinical specimens. J Clin Microbiol 2006; 44: 2698–2704. 16891480 10.1128/JCM.00542-06 PMC1594621

[bib27] Pallansch MA, Oberste MS, Whitton JL. Enteroviruses: polioviruses, coxsackieviruses, echoviruses, and newer enteroviruses. In: Knipe DM, Peter MH (eds). Fields Virology. 6th ed. Philadelphia: Lippincott Williams & Wilkins Press, 2013: 494–495.

[bib28] Xiang Z, Li L, Lei X et al. Enterovirus 68 3C protease cleaves TRIF to attenuate antiviral responses mediated by Toll-like receptor 3. J Virol 2014; 88: 6650–6659. 24672048 10.1128/JVI.03138-13 PMC4054379

[bib29] World Health Organization (WHO)Guidelines for WHO/EPI collaborative studies on poliomyelitis. Standard procedure for determining immunity to poliovirus using the microneutralization test. World Health Organization: Geneva. 1993. Available at http://www.who.int/iris/handle/10665/70486.

[bib30] Reed LJ, Muencha H. Simple method of estimating fifty percent endpoints. Am J Epidemiol 1938; 27: 493–497.

[bib31] Zhang Y, Moore DD, Nix WA et al. Neutralization of Enterovirus D68 isolated from the 2014 US outbreak by commercial intravenous immune globulin products. J Clin Virol 2015; 69: 172–175. 26209401 10.1016/j.jcv.2015.06.086 PMC6512324

[bib32] Smura T, Ylipaasto P, Klemola P et al. Cellular tropism of human enterovirus D species serotypes EV-94, EV-70, and EV-68 *in vitro*: implications for pathogenesis. J Med Virol 2010; 82: 1940–1949. 20872722 10.1002/jmv.21894

